# Concomitant, sequential, and 7-day triple therapy in first-line treatment of *Helicobacter pylori* infection in Korea: study protocol for a randomized controlled trial

**DOI:** 10.1186/s13063-017-2281-0

**Published:** 2017-11-17

**Authors:** Hyuk Lee, Beom Jin Kim, Sang Gyun Kim, Jin Il Kim, Il Ju Choi, Yong Chan Lee, Jae G. Kim, Jae J. Kim, Korean College of Helicobacter and Upper Gastrointestinal Research

**Affiliations:** 1 Department of Medicine, Samsung Medical Center, Sungkyunkwan University School of Medicine, 81 Irwon-ro, Gangnam-gu, Seoul 06351 South Korea; 2 Department of Internal Medicine, Chung-Ang University College of Medicine, 102 Heukseok-ro, Dongjak-gu, Seoul 06973 South Korea; 3 Department of Internal Medicine and Liver Research Institute, Seoul National University College of Medicine, Seoul, South Korea; 4 Department of Internal Medicine, The Catholic University of Korea College of Medicine, Seoul, South Korea; 5 Center for Gastric Cancer, National Cancer Center Hospital, National Cancer Center, Goyang, Gyeonggi South Korea; 6 Department of Internal Medicine, Institute of Gastroenterology, Yonsei University College of Medicine, Seoul, South Korea

**Keywords:** *Helicobacter pylori*, Triple therapy, Concomitant therapy, Sequential therapy

## Abstract

**Background:**

Most international guidelines recommend triple-therapy regimens consisting of a proton pump inhibitor, clarithromycin, and amoxicillin/metronidazole for at least 7 days for the eradication of *Helicobacter pylori*. However, the efficacy of 7-day clarithromycin-based standard triple therapy for *H. pylori* infection is currently unacceptable in Korea. In this study, we will compare the efficacy and safety of 7-day standard triple therapy, 10-day sequential therapy, and 10-day concomitant therapy for the first-line treatment of *H. pylori* infection in Korea.

**Methods/design:**

In this multicenter, investigator-blinded, randomized trial we are recruiting adult patients with *H. pylori* infection from 15 hospitals in Korea to determine whether sequential or concomitant treatment is superior to standard triple therapy. Patients are randomly assigned to receive either standard triple therapy (lansoprazole, amoxicillin, and clarithromycin) for 7 days, or sequential treatment (lansoprazole and amoxicillin for the first 5 days, followed by lansoprazole, clarithromycin, and metronidazole for another 5 days) for 10 days, or concomitant therapy (lansoprazole, amoxicillin, clarithromycin, and metronidazole) for 10 days. The primary outcome is the rate of *H. pylori* eradication in the intention-to-treat population.

**Discussion:**

The results of this study will be crucial for determining the optimal regimen for the primary treatment of *H. pylori* infection in Korea. This study will produce vital evidence that will lead to revisions to guidelines concerning first-line treatment regimens for *H. pylori* infection.

**Trial registration:**

Clinical Research Information Service (CRIS), Republic of Korea, KCT0001980. Registered on 25 July 2016.

**Electronic supplementary material:**

The online version of this article (doi:10.1186/s13063-017-2281-0) contains supplementary material, which is available to authorized users.

## Background

*Helicobacter pylori* infection causes peptic ulcers, gastric mucosa-associated lymphoid tissue lymphoma, and gastric cancer [1]. Most international guidelines recommended triple-therapy regimens consisting of a proton pump inhibitor (PPI), clarithromycin, and amoxicillin/metronidazole for at least 7 days for the eradication of *H. pylori* [2–4]. However, the eradication frequency of standard triple therapy has fallen to less than 80% in many countries because of the rising prevalence of clarithromycin resistance [5–7]. The rate of clarithromycin resistance is reported to be as high as 37.3% in Korea. Consequently, the first-line eradication rate of triple therapy has been shown to be 74% according to a national database [8, 9]. Despite this issue, the standard triple-therapy regimen is still recommended for first-line therapy in Korea according to the revised guidelines, and it is the only regimen reimbursed by the Korean National Health Insurance Service [10].

Several strategies, including bismuth-containing quadruple therapy and non-bismuth-containing quadruple therapy (either sequential or concomitant), have been proposed to increase the eradication rate in Korea [11–14]. Because the efficacy of sequential treatment, which consists of a PPI and amoxicillin for the first 5 days, followed by a PPI plus clarithromycin and metronidazole for another 5 days, appears to be less susceptible to clarithromycin resistance than triple therapy, it was expected to become the standard first-line treatment for *H. pylori* infection. A meta-analysis indicated that sequential therapy was more effective than 7-day triple therapy, but there are regional differences in the efficacy of this approach, owing to geographical variations in antibiotic resistance [15, 16]. Furthermore, the pooled eradication rates of sequential therapy as a first-line treatment are reported to be suboptimal in Korea [17]. Nonbismuth quadruple therapy is another alternative to concomitant therapy, and it is proven to work even when clarithromycin resistance is present [18]. Several randomized trials comparing sequential with concomitant treatment have indicated no significant differences, whereas sequential therapy was found to be slightly more effective in Central and South America [19, 20]. In a small number of randomized controlled studies in Korea, 10-day or 5-day concomitant therapy was shown to be more effective than standard triple therapy [21, 22]. Some retrospective studies have demonstrated that concomitant therapy is superior to standard triple therapy [23]. Concomitant and sequential therapies were demonstrated to be similarly effective in a few head-to-head comparison studies in Korea [13]. Despite these previous results, evidence supporting these conclusions is insufficient because no studies with an adequately rigorous design have been performed. With these considerations in mind, we designed the present nationwide, population-based, randomized controlled trial to compare the efficacy of 10-day concomitant therapy, 10-day sequential therapy, and 7-day triple therapy as first-line therapy for *H. pylori* infection in a Korean population.

## Methods/design

### Trial design and study setting

This study is designed as a multicenter, randomized, three-arm, superiority, parallel-group trial. Participants will be randomly allocated to the following three arms in a 1:1:1 ratio:

1. *Arm 1 (control arm):* The standard triple-therapy group will receive lansoprazole 30 mg, amoxicillin 1 g, and clarithromycin 500 mg twice daily for 7 days.
2. *Arm 2 (experimental arm 1):* The sequential therapy group will receive lansoprazole 30 mg and amoxicillin 1 g twice daily for the first 5 days, followed by lansoprazole 30 mg, clarithromycin 500 mg, and metronidazole 500 mg twice daily for the remaining 5 days.
3. *Arm 3 (experimental arm 2):* The concomitant therapy group will receive lansoprazole 30 mg, amoxicillin 1 g, clarithromycin 500 mg, and metronidazole 500 mg twice daily for 10 days.

**Fig. 1:**
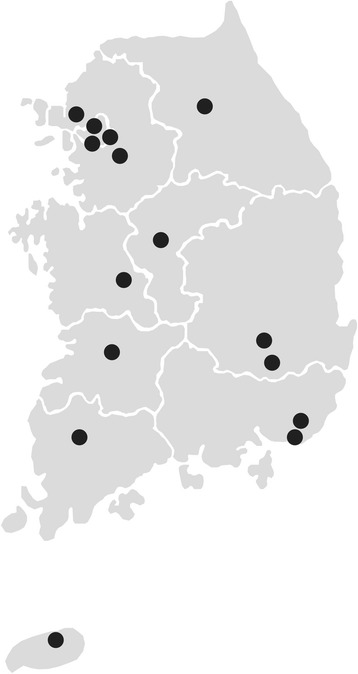
Map showing the geographic locations of the 15 participating study centers in Korea

**Fig. 2:**
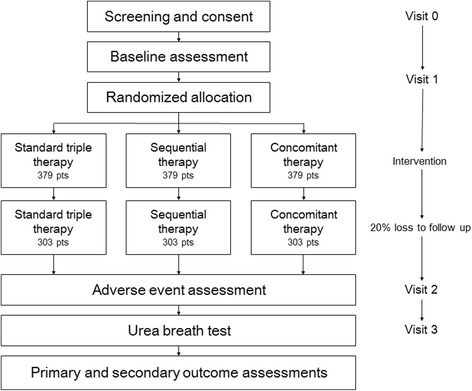
Study design

Participants will be recruited from 15 hospitals in Korea (Samsung Medical Center, Chung-ang University Hospital, Yonsei University Severance Hospital, National Cancer Center, Bundang Seoul National University Hospital, Chungbuk National University Hospital, Chungnam National University Hospital, Chonnam National University Hospital, Chonbuk National University Hospital, Chilgok Kyungpook National University Hospital, Keimyung University Dongsan Hospital, Pusan National University Hospital, Kosin University Gospel Hospital, Jeju National University Hospital, and Hallym University Chuncheon Sacred Heart Hospital) (Fig. 1). The recruitment period began in November 2016 and will end in October 2018. We received approval to conduct this study from the institutional review boards of 15 nationwide hospitals. This investigator-initiated trial was approved by the Korean Food and Drug Administration and is registered at https://cris.nih.go.kr/cris (KCT0001980). A flow diagram of the trial is shown in Fig. 2.

### Study subjects

Consecutive outpatients with *H. pylori* infection aged ≥ 19 years who have undergone endoscopic examination within the prior 3 months, agree to trial participation, and provide written informed consent fulfill the eligibility criteria and are invited to participate in the study. Exclusion criteria include a history of *H. pylori* eradication therapy; gastric surgery; abnormal liver function test or renal function test results on screening laboratory studies; antibiotic use within the prior 4 weeks; PPI use within the prior 2 weeks; histamine-2 receptor antagonist, aspirin, nonsteroidal anti-inflammatory drug, steroid, anticholinergic, prostaglandin analog, promotility drug, or sucralfate use within the prior week; use of drugs such as lovastatin, simvastatin, atorvastatin, indinavir, ritonavir, cyclosporin, terfenadine, cisapride, pimozide, astemizole, HIV protease inhibitors, and ergot alkaloids; allergic reactions to the study medications; and serious concomitant illnesses. In addition, we will exclude pregnant participants or participants who plan to become pregnant as well as patients participating in other clinical studies or who are otherwise deemed unsuitable by the researchers. Withdrawal criteria include protocol violations such as detection of eligibility violations, use of any forbidden medication during the trial that could influence the study results, or occurrence of other significant protocol violations. In addition, subjects with serious adverse events, an allergic reaction to the investigational product, withdrawal of consent, and decision to terminate for reasons of the subject’s health will be withdrawn from the study. Symptomatic patients will be allowed to use antacids on demand in the posttreatment period. Antibiotics or other medications affecting the treatment results will be prohibited during the study period.

### Randomization and masking

Randomization will be conducted using a centralized, web-based randomization system managed by the Medical Research Collaborating Center (https://mrcc.snuh.org) of Seoul National University Hospital, which uses permuted block randomization with a concealed and varying block size. Only the researcher at the Medical Research Collaborating Center is able to know which number indicates which treatment arm. To ensure concealed allocation, an independent staff member will dispense consecutively numbered, identically designed treatment packs that contain sealed bottles of study drugs. Physicians who are unaware of treatment assignment will manage all study participants. Participants will not be blinded to group allocation.

### Procedures

**Fig. 3:**
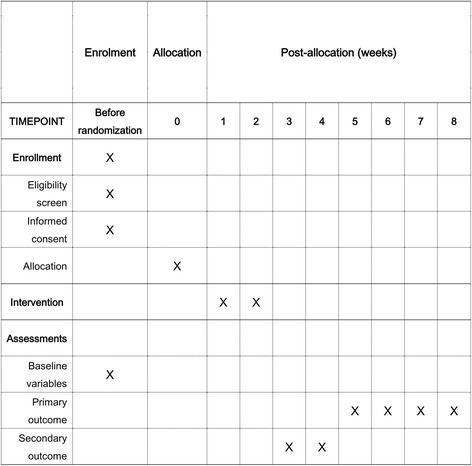
Standard Protocol Items: Recommendations for Interventional Trials (SPIRIT) figure

The standard protocol for this study is shown in Fig. 3. Before enrollment, *H. pylori* infection status will be determined by a rapid urease test, urea breath test, histology, and/or bacterial culture. The patients will complete a standard questionnaire to collect complete demographic data, including age, sex, medical history, family history, history of smoking and alcohol consumption, and gastrointestinal symptoms. The patients will receive a printed handout that provides directions for taking the medications correctly and how best to adhere to treatment. The follow-up period of this study after randomization will be 8 weeks and will include two visits with each subject (first visit at 3 or 4 weeks after allocation, second visit 5–8 weeks after allocation). Adverse events and compliance will be evaluated at the first visit after allocation. At the second visit, the efficacy of *H. pylori* eradication therapy will be determined. In all subjects, a urea breath test will be conducted to assess *H. pylori* status at the fourth to sixth week after the end of *H. pylori* eradication therapy. A staff member blind to the eradication arm of each patient will perform the urea breath tests. The cutoff values will be set at 2% for the capsule urea breath test and 4.8% for the conventional urea breath test. Eradication will be defined as a negative result for the urea breath test. A Standard Protocol Items: Recommendations for Interventional Trials (SPIRIT) checklist is provided as an additional file (Additional file 1).

### Tolerability and adherence

Drug tolerance and patient compliance will be evaluated at the end of treatment. Drug tolerance will be investigated by questioning patients about possible side effects, such as nausea, vomiting, dry mouth, diarrhea, constipation, rash, dizziness, disorientation, and candidiasis. Before treatment, the patients will be informed of the common adverse events associated with the study drugs and will be asked to record these symptoms during treatment in provided diaries. The adverse events will be assessed according to a 4-point scale: none, mild (discomfort annoying but not interfering with daily life), moderate (discomfort sufficient to interfere with daily life), and severe (discomfort resulting in discontinuation of eradication therapy) [24]. Incapacitating or life-threatening complications will be classified as serious and will be reported to a clinical research associate. Adherence to treatment will be assessed by providing all patients with a prestructured printed table with all dosages illustrated and then asking them to tick each time a pill was consumed and bring back the table along with any tablet not consumed, which will be counted. In cases of discrepancies between the structured printed table and residual medication, the latter will be taken into account to evaluate the patient’s adherence. Good compliance will be defined as the use of at least 80% of the total medication prescribed.

### Data management

The electronic case report form development and data management for this study will be performed using the Internet-based Clinical Research and Trial (iCReaT) management system (http://icreat.nih.go.kr), which is a data management system established by the Centers for Disease Control and Prevention, Ministry of Health and Welfare, Republic of Korea (iCReaT study number C160025).

### Monitoring

Because the known risks are minimal in this trial, no official data monitoring committee will be established in each study institution. Instead, to maintain the quality of this clinical trial, the clinical research associate will conduct regular monitoring by reviewing the informed consent form, case report form, investigator study file, compliance, serious adverse event, and data records. Monitoring will begin after the first participant completes the whole process of this study. Every institution where the trial is being conducted will be monitored using standard operating procedures while the trial is in process.

### Sample size

Several studies have demonstrated that the eradication rate of standard triple therapy is approximately 75% in Korea [25]. The eradication rate of sequential or concomitant therapy has been reported to be more than 80% [23, 26]. To obtain the optimal eradiation rate, an efficacy greater than 85% is needed for each treatment [27]. Therefore, we hypothesized that the eradication rate would be superior in the sequential or concomitant therapy groups, with a 10% difference compared with the rate for standard triple therapy (85% vs. 75%). To demonstrate this 10% difference in the eradication rate using a statistical power of 80% and with the assumption of a one-sided error rate of 0.025, the protocol requires at least 909 randomly assigned participants. The sample size in this trial was estimated according to the following formula:

$$ n=\frac{{\left\{{\mathrm{z}}_{\alpha}\surd 2P\left(1-P\right)+{\mathrm{z}}_{\beta}\surd {P}_1\left(1-{P}_1\right)+{P}_2\left(1-{P}_2\right)\right\}}^2}{{\left({P}_1-{P}_2\right)}^2} $$

To allow for possible dropouts, defined as patients who fail to present or to follow the medication instructions, 1137 subjects will be required.

### Statistical analysis

All efficacy analyses will be calculated for the intention-to-treat (ITT) and per-protocol (PP) populations. The ITT analysis will include all randomized patients who take at least one dose of the study medication. The patients whose infection status is unknown following treatment will be considered as treatment failures for the purposes of ITT analysis. The PP analysis will exclude patients with unknown *H. pylori* status following therapy and those with major protocol violations. The chi-square test, analysis of variance (ANOVA), or Fisher’s exact test, as appropriate, will be performed to compare demographic and baseline characteristics between treatment groups. The number and percentage of compliant patients will be tabulated according to the treatment regimen. Differences in compliance rates among treatment regimens will be assessed with 95% CIs. The chi-square test, ANOVA, or Fisher’s exact test, as appropriate, will be performed to compare adverse events and laboratory parameters between groups. The primary endpoint of the study is the *H. pylori* eradication rate after first-line treatment. The secondary endpoints are the frequency of adverse events, treatment compliance, and gastrointestinal symptomatic response after eradication.

### Ethics and dissemination

The study is being conducted in accordance with Declaration of Helsinki standards and Food and Drug Administration regulations regarding good clinical practice. All participants will have been informed of the possible risks and benefits of participating in this clinical trial. After being provided sufficient time to ask questions, participants will be provided an informed consent form that was approved by the local institutional review board. A signed copy of the consent form will be maintained with the study records. Trial participants will be provided a unique participant identification number to maintain confidentiality, and no protected health information will be disseminated. Trial results will be disseminated through scientific conference presentations and by publication in scientific journals.

## Discussion

The Korean College of Helicobacter and Upper Gastrointestinal Research proposed revised guidelines in 2013 [10, 28]. These revised guidelines recommend standard triple therapy including a conventional PPI, clarithromycin, and amoxicillin as the primary eradication regimen with a high level of evidence and strong recommendation grade. Furthermore, only this regimen is reimbursed by the Korean government and health insurance system.

However, a recent study using a nationwide Korean database demonstrated that the eradication rate of clarithromycin-based standard triple therapy is 73% [9]. Furthermore, a comprehensive meta-analysis of Korean reports indicated an overall eradication rate of 74.6% by ITT analysis and 82.0% by PP analysis [29]. More importantly, the eradication rate has decreased significantly over the past 10 years [30]. However, this decreasing trend was reported to be different in subgroup analysis, depending on the geographic area. This appeared to be caused by the amount of macrolide antibiotic used [30]. In this respect, clinical guidelines for *H. pylori* eradication should be modified on the basis of results of a nationwide trial, given the variation in eradication rates after standard triple therapy in each region. Most randomized studies involving the efficacy of first-line treatment were conducted at a single center, and very few multicenter studies have been published to date [29]. A recent nationwide randomized trial revealed that sequential therapy provides higher eradication rates than does standard triple therapy as a first-line therapy in Korea, with ITT eradication rates of 70.8% for standard triple therapy and 82.4% for sequential therapy [26]. However, as the authors mentioned, there was a lack of uniformity in the methodologies used among different institutions.

The efficacy of sequential or concomitant therapy for first-line treatment has been reported to be superior to that of clarithromycin-based standard triple therapy by many Korean researchers, which is in agreement with data from other countries. Nevertheless, a pooled analysis showed that sequential treatment led to suboptimal eradication rates in a Korean population, perhaps due to high rates of resistance to clarithromycin and metronidazole [19]. Hence, regional variations in antibiotic resistance need to be understood so that the global optimal protocol for different Korean regions can be determined.

This study is designed to assess the two regimens of greatest interest at present. Furthermore, we will enroll patients from across the different regions of Korea, and a sufficient sample size will be obtained to answer the study question in a statistically rigorous manner. This study will produce vital evidence that will lead to revisions to guidelines concerning first-line treatment regimens for *H. pylori* infection, better eradication rates, and an improved patient quality of life.

### Trial status

Recruitment is ongoing. The first patient was randomly assigned to a treatment group in November 2016. Patient enrollment reached 75% of target number.

## Additional file

Additional file 1: SPIRIT 2013 checklist: recommended items to address in a clinical trial protocol and related documents. (TIF 3398 kb)

## Abbreviations

ANOVA: Analysis of variance
iCReaT: Internet-based Clinical Research and Trial management system
ITT: Intention to treat
PP: Per protocol
PPI: Proton pump inhibitor
SPIRIT: Standard Protocol Items: Recommendations for Interventional Trials
