# Correction to: Concomitant, sequential, and 7-day triple therapy in first-line treatment of *Helicobacter pylori* infection in Korea: study protocol for a randomized controlled trial

**DOI:** 10.1186/s13063-017-2393-6

**Published:** 2017-12-15

**Authors:** Hyuk Lee, Beom Jin Kim, Sang Gyun Kim, Jin Il Kim, Il Ju Choi, Yong Chan Lee, Jae G. Kim, Jae J. Kim, Korean College of Helicobacter and Upper Gastrointestinal Research

**Affiliations:** 1 Department of Medicine, Samsung Medical Center, Sungkyunkwan University School of Medicine, 81 Irwon-ro, Gangnam-gu, Seoul 06351 South Korea; 2 Department of Internal Medicine, Chung-Ang University College of Medicine, 102 Heukseok-ro, Dongjak-gu, Seoul 06973 South Korea; 3 Department of Internal Medicine and Liver Research Institute, Seoul National University College of Medicine, Seoul, South Korea; 4 Department of Internal Medicine, The Catholic University of Korea College of Medicine, Seoul, South Korea; 5 Center for Gastric Cancer, National Cancer Center Hospital, National Cancer Center, Goyang, Gyeonggi South Korea; 6 Department of Internal Medicine, Institute of Gastroenterology, Yonsei University College of Medicine, Seoul, South Korea

## Correction

In the original publication [1] was an error in the grant number in the funding section. The correct version can be found in this Erratum.

Incorrect version:

**Funding**

This research was supported by a grant from the Korea Health Technology R&D Project through the Korea Health Industry Development Institute (KHIDI), funded by the Ministry of Health & Welfare, Republic of Korea (grant number HI15C1234).

Correct version:

**Funding**

This research was supported by a grant from the Korea Health Technology R&D Project through the Korea Health Industry Development Institute (KHIDI), funded by the Ministry of Health & Welfare, Republic of Korea (grant number HC15C1077).

## References

[1] Lee H. Concomitant, sequential, and 7-day triple therapy in first-line treatment of *Helicobacter pylori* infection in Korea: study protocol for a randomized controlled trial. Trials. 2017;18:549. doi: 10.1186/s13063-017-2281-0 10.1186/s13063-017-2281-0 PMC5693582 29149904

